# Mechanisms of Activation of Receptor Tyrosine Kinases: Monomers or Dimers

**DOI:** 10.3390/cells3020304

**Published:** 2014-04-22

**Authors:** Ichiro N. Maruyama

**Affiliations:** Information Processing Biology Unit, Okinawa Institute of Science and Technology Graduate University, 1919-1 Tancha, Onna-son, Kunigami, Okinawa 904-0495, Japan; E-Mail: ichi@oist.jp; Tel.: +81-98-966-8496; Fax: +81-98-966-2890

**Keywords:** BDNF, cancer, dimerization, EGFR, IGF, ligand, NGF, phosphorylation, rotation/twist, transmembrane signaling, Trk

## Abstract

Receptor tyrosine kinases (RTKs) play essential roles in cellular processes, including metabolism, cell-cycle control, survival, proliferation, motility and differentiation. RTKs are all synthesized as single-pass transmembrane proteins and bind polypeptide ligands, mainly growth factors. It has long been thought that all RTKs, except for the insulin receptor (IR) family, are activated by ligand-induced dimerization of the receptors. An increasing number of diverse studies, however, indicate that RTKs, previously thought to exist as monomers, are present as pre-formed, yet inactive, dimers prior to ligand binding. The non-covalently associated dimeric structures are reminiscent of those of the IR family, which has a disulfide-linked dimeric structure. Furthermore, recent progress in structural studies has provided insight into the underpinnings of conformational changes during the activation of RTKs. In this review, I discuss two mutually exclusive models for the mechanisms of activation of the epidermal growth factor receptor, the neurotrophin receptor and IR families, based on these new insights.

## 1. Introduction

Phosphorylation of tyrosine is a key post-translational modification of proteins in the propagation of extracellular information to intracellular signal transduction. Receptor tyrosine kinases (RTKs) function through the protein kinase domain located in the intracellular region of each RTK monomer. Ligand binding to the extracellular region results in the elevation of the receptor’s tyrosine kinase activity and in selective *trans*-autophosphorylation of tyrosine residues. Some of these sites are involved in maintaining the active conformation of the kinase itself, while others become docking sites for various adaptor/effector scaffold proteins and enzymes. 

**Figure 1:**
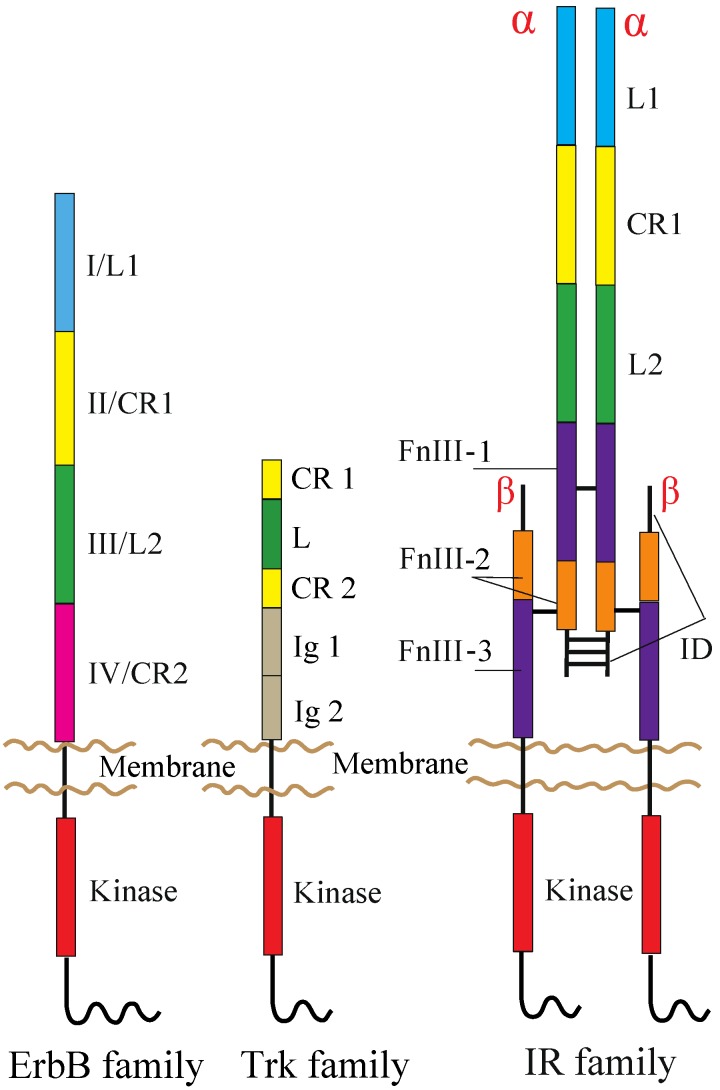
Domain organization of RTKs. The following abbreviations are used: L, leucine-rich; CR, cysteine-rich; Ig, immunoglobulin-like; FnIII, fibronectin type III; ID, insert domain. The L1, CR1, L2 and CR2 domains of the ErbB family are alternatively termed Domains I–IV. The ErbB and Trk families are drawn as a monomer, but might be present as non-covalently formed dimers at the cell surface prior to ligand binding (see the main text). Not drawn to scale.

The human RTK superfamily consists of 58 proteins grouped into 20 sub-families [1]. Apart from the insulin receptor (IR) subfamily, RTKs are all expressed as single protomers that form non-covalently associated dimers. The IR family, consisting of IR, the insulin-like growth factor I-receptor (IGF-IR) and the insulin receptor-related receptor (IRR), is also expressed as a single subunit, but it undergoes processing into form two, α and β, polypeptide chains that are assembled into a heterotetramer, or an (αβ)_2_ homodimer, stabilized by disulfide bonds (Figure 1).

RTK protomers are integral membrane proteins, and their N-terminal extracellular regions are generally composed of various structural modules with multiple, intrachain disulfide bonds and numerous N-linked glycosylation sites. The extracellular region is joined to the intracellular region by a transmembrane (TM) segment. The intracellular region has a tyrosine kinase domain flanked by an intracellular juxtamembrane (JM) region and a C-terminal tail (or extension). The JM and C-terminal tail regions differ in size and tyrosine content among family members, and these differences generate and propagate different intracellular signals.

The total number of tyrosine residues found in each intracellular region and the number known to be modified differ significantly between superfamily members, and the distribution of tyrosine residues is quite variable [2,3]. For example, the intracellular region of the epidermal growth factor receptor (EGFR), which is a member of the ErbB receptor family (originally named because of the homology to the erythroblastoma viral gene product, v-erbB), has 20 tyrosine residues, 12 of which are known to be phosphorylated, while the intracellular region of TrkA, a member of the neurotrophin (NT) receptor family, contains 11 tyrosine residues, six of which can be phosphorylated. One common modification in the RTK superfamily is the phosphorylation of tyrosine(s) in the activation loop of the kinase domain. An exception to this rule among the commonly studied members of the superfamily is EGFR. It has only a single tyrosine in this position, which is not required for kinase function, although it is phosphorylated upon activation by the epidermal growth factor (EGF) [4]. The role of the activation loop tyrosine(s) is to stabilize the loop in an open conformation, so that both ATP and the substrate peptide can be bound [5]. 

Other phosphotyrosines provide binding sites for soluble or membrane-anchored proteins that are recruited upon receptor activation [6]. These include scaffolding proteins, which provide additional docking sites for effectors. RTKs activate several pathways linked to cellular phenotypic responses, and these have been extensively studied [7,8]. These include signaling cascades mediated by Ras/Raf/MAP kinase, phosphoinositide-3-kinase (PI3K)/Akt and phospholipase Cγ (PLC-γ). 

It has long been thought that ligand binding activates RTKs by inducing receptor dimerization [9,10,11,12,13,14]. However, an increasing number of studies demonstrate that RTKs exist as pre-formed, yet inactive, dimers, even in the absence of activating ligand [15,16,17,18,19,20,21,22,23,24,25,26,27,28,29]. IR and IGF-IR are also expressed as disulfide-linked (αβ)_2_ homodimers at the cell surface [30]. The ErbB receptor, neurotrophin (NT) receptor and IR families are among the RTKs most extensively studied. Recent crystallographic studies on these receptors with and without bound ligand have led to a clearer understanding of the molecular structures of the RTK active and inactive forms. Negative cooperativity was also observed both in EGFR-EGF and IR-insulin interactions, suggesting that the underlying mechanisms of activation of these receptors by ligand binding may be similar. This review article specifically focuses on the molecular mechanisms of the receptor activation based on the structures elucidated.

## 2. RTKs and Their Ligands

### 2.1. ErbB Family

EGF was discovered in 1962 [31], and its interaction with a cell surface receptor was first characterized in 1975 [32]. The ErbB family consists of EGFR (also known as ErbB1 or HER1), ErbB2 (HER2/Neu), ErbB3 (HER3) and ErbB4 (HER4), and plays important roles in cell growth, differentiation, survival and migration [33,34]. Each ErbB receptor is essential for normal animal development [35]. Aberrant activation of ErbB receptors is implicated in a variety of human cancers [36]. Loss of ErbB4 function results in defects in the heart, nervous system and mammary gland in mice [37,38]. The EGF family binds ErbB receptors, and on the basis of their receptor specificity, they are divided into three groups. The first group includes EGF, transforming growth factor (TGF)-α, amphiregulin (AR) and epigen (EPG), which bind specifically to EGFR. The second group includes beta-cellulin (BTC), heparin-binding EGF (HB-EGF) and epiregulin (EPR), which exhibit dual specificity, binding both EGFR and ErbB4. The third group, the neuregulins (NRGs), forms two subgroups, depending on whether they bind ErbB3 and ErbB4 (NRG-1 and NRG-2) or only ErbB4 (NRG-3 and NRG-4) [39,40]. 

ErbB receptors consist of an extracellular ligand-binding region (~620 amino acid residues), a single TM segment (~25 residues), an intracellular JM region (~40 residues), a cytoplasmic kinase domain (~270 residues) and a 220–350 amino acid C-terminal tail that becomes tyrosine phosphorylated following activation and mediates interactions between ErbB receptors and downstream effectors. Mammalian ErbB receptor extracellular regions contain four distinct domains (I–IV) (Figure 1) [12,41,42]. Domains I (also known as L1) and III/L2 are both β-helix solenoid structures and share 37% sequence identity with EGFR. These domains are related to the leucine-rich repeat superfamily [43] and are responsible for ligand binding by simultaneously contacting the same bound ligand. Domains II/CR1 and IV/CR2 are both cysteine-rich domains with disulfide bonds similar to those seen in laminin and tumor necrosis factor (TNF) receptor [44]. IR and IGF-IR also share both types of the domains with the ErbB receptors (Figure 1) [30]. 

In contrast to EGFR, which recognizes a variety of different ligands, ErbB2 lacks a known ligand [45] and acts as a heterodimerization partner for each of the other ErbBs, irrespective of the stimulating ligand [46,47,48,49]. ErbB3 has been reported either to have no detectable kinase activity in studies using recombinant protein [50,51] or to have very low activity when immunoprecipitated from cells [52,53]. ErbB3 therefore must heterodimerize with other kinase-active ErbB receptors to signal, and ErbB2 appears to be the preferred dimerization partner for ErbB3 [47]. In the ErbB2/ErbB3 heterodimer, both ErbB2 and ErbB3 become phosphorylated [47]. As phosphorylation occurs *in trans*, it is clear that the kinase-active ErbB2 could phosphorylate the C-terminal tail of ErbB3. However, it is difficult to explain how ErbB2 could be phosphorylated by the kinase-inactive ErbB3 in the context of an ErbB2/ErbB3 heterodimer. Indeed, it has recently been shown that ErbB3 possesses sufficient kinase activity to robustly *trans*-autophosphorylate its own intracellular region [54]. In addition, phosphorylation of ErbB2 may also occur within ErbB2/ErbB3 tetramers, in which the ErbB2 from one dimer phosphorylates the ErbB2 in the other dimer [55]. 

### 2.2. NT Receptor Family

The pioneering discovery of the nerve growth factor (NGF) [56] set the stage for the discovery of other NTs. In mammals, NGF, brain-derived neurotrophic factor (BDNF), NT-3, NT-4/5, NT-6 and NT-7 act through the NT receptor family, which consists of three tropomyosin-related kinase receptors (TrkA, TrkB and TrkC) and p75^TNR^, which is a member of the TNF receptor superfamily and does not have kinase activity. TrkA preferentially interacts with NGF, NT-7, and to a lesser extent, NT-6 [57,58]. TrkB interacts with BDNF and NT-4/5 [59,60], and TrkC is specific for NT-3 [61,62,63]. NT-3 can also interact with TrkA and TrkB with low affinity, and all the NTs can bind p75^NTR^ with low affinity [64,65,66]. NTs are initially synthesized in the endoplasmic reticulum as prepro-NTs, and cleavage of the signal peptide of prepro-NTs converts these into pro-NTs. In the *trans*-Golgi network and in secretory vesicles, pro-NTs dimerize and are proteolytically processed by proprotein convertase enzymes to their mature forms before their release from the cell [67]. In the extracellular space, pro-NTs may be cleaved by plasmin, and the secreted, mature forms of NTs exist in solution as dimers [67,68]. 

Trk receptor kinases play crucial roles in the development and maintenance of the central and peripheral nervous systems, in the preventing or reversing of neuronal degeneration and in the enhancement of synaptic plasticity [69]. TrkA is widely expressed in sympathetic, trigeminal and dorsal root ganglia and in cholinergic neurons of the basal forebrain and striatum [58,70,71]. TrkB is found throughout the central and peripheral nervous systems, and TrkC is widely expressed in mammalian neural tissues [61,72,73]. Activation of Trk initiates downstream signaling cascades mediated by Ras/Raf/MAP kinase, PI3K/Akt and PLC-γ [74,75,76]. Aberrant activation of Trk kinases is often observed in human cancers. Constitutively active TrkA fusions occur in some thyroid cancers and colon carcinomas [77]. TrkB and its ligand, BDNF, are highly expressed in biologically unfavorable neuroblastomas, and TrkB expression is associated with drug resistance and the expression of angiogenic factors [78]. Fusion of the TrkC gene with the ETV6 transcription factor gene has been described in oncogenic carcinomas and acute myelogenous leukemias [79]. 

TrkA, TrkB and TrkC share significant sequence homology and a conserved domain organization comprising, from the N-terminus to C-terminus, an extracellular region, a TM segment and an intracellular region containing the kinase domain (Figure 1). The extracellular region consists of five domains, a leucine-rich region (L) flanked by two cysteine-rich regions (CR1 and CR2) and two immunoglobulin-like domains (Ig1 and Ig2) [80]. The kinase domains of TrkA, TrkB and TrkC share between 71.9% and 78.3% sequence identity, TrkB and TrkC being the closest homologues [81]. Studies on TrkB and TrkC have shown that the Ig2 domain is sufficient for the binding of ligands and is responsible for their binding specificity [82,83,84]. The crystal structures of Ig2 of TrkA, TrkB and TrkC, as well as TrkA Ig2 in complex with NGF have been solved [85,86].

### 2.3. IR Family

Insulin is a peptide hormone discovered in 1921 [87], and its ability to promote glucose uptake into tissues was demonstrated in 1949 [88]. Insulin, IGF-I and IGF-II share a common three-dimensional architecture and bind IR and IGF-IR with differing affinities. In contrast to the critical role of insulin in metabolic control, the IGFs act via IGF-IR to promote cell proliferation, survival and differentiation. IGFs are essential for normal growth and development, and perturbation of IGF-I expression is associated with acromegaly [89] or short stature [90]. Disruption of IGF-II imprinting during development is associated with overgrowth in Beckwith–Wiedemann syndrome, whereas reduced paternal allele expression results in growth retardation in Silver–Russell syndrome [91]. Furthermore, IGFs acting via the IGF-IR play a major role in promoting cancer cell growth and survival [92]. 

Specific cell-surface receptors for insulin were identified in 1971 [93]. The IR exists in two isoforms, IR-A and IR-B, which arise by alternative splicing of exon 11 [94,95]. The IR-B isoform, which differs from IR-A by the presence of a 12-residue segment (encoded by exon 11) inserted between IR-A Residues 716 and 717, three residues before the C-terminus of the α-chain, binds insulin with high affinity. The IR-A isoform can also binds IGF-II, albeit with a six-fold lower affinity [96,97]. These receptors are disulfide-linked homodimers, which also function as heterodimer hybrids, since IR::IGF-IR hybrids have been detected in all tissues that express both receptors [98,99]. While stimulation of IR with insulin primarily modulates cellular metabolism, the main function of activated IGF-IR is to promote cell proliferation and survival [100,101]. None of the known IR or IGF-IR ligands can activate IRR [102,103], which is primarily expressed in the kidneys, stomach and pancreas [104,105]. Recently, IRR has been shown to be activated by alkaline media both *in vitro* and *in vivo* at pH > 7.9, indicating its role as an alkaline sensor molecule in the kidney [106]. A triple *Ir* *Igf1r* *Irr* gene knockout fails to develop the male phenotype, while all single and double knockouts do, suggesting that IRR can substitute for the other receptors in mice and is required for male sexual differentiation [107].

Each receptor extracellular region consists of, from the N-terminus to the C-terminus, a leucine-rich repeat domain (L1), a cysteine-rich region (CR), a second leucine-rich repeat domain (L2) and three fibronectin type III domains (FnIII-1, FnIII-2 and FnIII-3), the second of which contains the large (~120 residues) insert domain (ID) (Figure 1) [108,109]. The ID contains a furin cleavage site that generates the α-chain and β-chain of the mature receptor monomer. The intracellular region of each IR family monomer contains a tyrosine kinase domain flanked by two regulatory regions, the JM region and the C-terminal tail, that contain the phosphotyrosine binding sites for effector signaling molecules [110]. In particular, the JM region is involved in docking IR substrates, IRS1-4 [111] and Shc, as well as in receptor internalization [112,113,114]. Activation of IR and IGF-IR leads to signaling via two main pathways. Following the activation of the tyrosine kinase domain, receptors undergo autophosphorylation, which promotes the binding of effector molecules. These proteins then lead to the activation of PI3K/Akt and the extracellular signal-regulated kinase (ERK/MAPK) cascades [113]. 

## 3. Are TRKs Monomeric or Dimeric Prior to Ligand Binding?

### 3.1. ErbB Family

EGFR is one of the first receptors for which ligand-induced dimerization was proposed as a primary event in transmembrane signaling, mainly based on biochemical data [9,115,116]. In this ‘dimerization model’, the ErbB family receptor is thought to exist as a monomer at the cell surface prior to ligand binding, and ligand binding induces dimerization, as a result of which intracellular kinase domains become closer and *trans*-phosphorylate each other. Furthermore, recently, it has been suggested that the receptor monomers are at equilibrium with the receptor dimers [8,117]. A limited population of receptor dimers (<10% of total receptors) exists with quaternary structures of their extracellular and cytoplasmic regions in configurations that are compatible with *trans*-autophosphorylation (active dimer). Ligand binding to the extracellular region stabilizes the formation of active dimers and, consequently, induces kinase stimulation [8]. 

More recently, an increasing number of studies have demonstrated that prior to ligand binding, ErbB receptors exist as dimers at the cell surface [15,16,17,18,19,20,21,22,23,24,25,28,29]. By chemical cross-linking, it was found that >80% of EGFR is dimeric in the absence of bound ligand [15]. Hetero-Förster resonance energy transfer (FRET) [16,18,20], fluorescence correlation spectroscopy (FCS) [20,21,25] and homo-FRET [23] analyses further demonstrated that EGFR and ErbB2 are present as dimers at physiological expression levels at the surface of living cells. Fluorescent protein fragment complementation indicates that the majority, if not all, of all the members of the ErbB family exists as dimers in living cells [22]. This is consistent with the results from reversible firefly luciferase enzyme fragment complementation analysis that all the EGFR and ErbB3 receptors exist as dimers in living cells, since fluorescence intensity was never increased by the addition of EGF to the cell culture [24,28,29]. Single molecule observation using total internal reflection fluorescence (TIRF) microscopy using oblique illumination also indicates the existence of dimers in living cells [19]. Depending on the methods used, dimer-to-monomer ratios vary from 40%–100%. Considering inefficient chemical crosslinking [118] and inefficient folding of fluorescent proteins [119,120,121], these ratios are likely to be, if at all, underestimated. The existence of significant numbers of pre-formed dimers is supported by evidence that autophosphorylation of the receptors can be enhanced by inhibitors of protein tyrosine phosphatases or by receptor overexpression, even in the absence of bound ligand [8,122]. 

However, this has recently been challenged. Brightness analysis of ErbB receptors using fluorescence microscopy suggested that at lower expression levels than 2 × 10^5^ molecules per cell, EGFR is monomeric [123]. Since the brightness of fluorescent protein tags differs in cytosol and close to the cell membrane [20], the existence of monomers should be re-evaluated based on the brightness of reference monomeric molecules in the vicinity of the membrane. Furthermore, a recent study that used two-color pulsed-interleaved excitation fluorescence cross-correlation spectroscopy, in which a pair of lasers alternatively excited GFP and mCherry with subnanosecond pulses, to analyze if EGFR expressed at very low levels in COS-7 cells, found that it is present as a monomer at the surface of living cells [14]. Since COS-7 expresses low levels of endogenous EGFR [124], however, the result should be re-evaluated using cells that do not express endogenous EGFR. 

### 3.2. NT Receptor Family

It is also under debate whether the Trk receptors are monomeric, dimeric or oligomeric prior to ligand binding. NT has been proposed to induce Trk dimerization [125,126], whereas there is evidence that Trk receptors exist as pre-formed dimers [26,27] or oligomers [127] at the cell surface prior to NT binding. In the absence of bound NTs, p75^NTR^ has a disulfide-linked dimeric structure through a highly conserved cysteine in its TM segment [128]. A recent biophysical analysis based on the diffusivity of receptors at the surface of the membrane suggests that >70% and ~20% of TrkA molecules are monomeric and dimeric or oligomeric, respectively [129]. It is, however, necessary to correlate the diffusivities with monomeric, dimeric and oligomeric structures by other means, since TrkA may exist as a pre-formed dimer with the highest diffusion rate, and a small fraction of the receptors may be present as tetramers or oligomers, possibly near coated pits after autophosphorylation, due to the receptor’s overexpression. Although functional interactions between Trk receptors and p75^NTR^ are apparent, the nature of their physical associations and the formation of complexes with NTs remain areas of ongoing debate and study [130,131,132,133].

### 3.3. IR Family

Unlike other RTKs, IR and IGF-IR are covalently disulfide-linked (αβ)_2_ dimers made of two extracellular α-subunits that contain the ligand-binding domains and two transmembrane β-subunits that contain the intracellular kinase domain [134,135,136]. There is evidence for the existence of a covalently disulfide-linked (αβ)_2_ hybrid dimeric receptor (IR::IGF-IR), which is composed of an IR αβ hemireceptor and an IGF-IR αβ hemireceptor [137,138,139,140]. Cross-talk between insulin, IGFs and their receptors appears to be relatively common in many tissues and organs. Like insulin, IGF-I also exhibits important metabolic effects, for example *in vivo* infusion of recombinant IGF-I leads to an acute decrease in circulating glucose values [141]. 

## 4. Mechanisms of RTK Activation

### 4.1. ErbB Family

**Figure 2:**
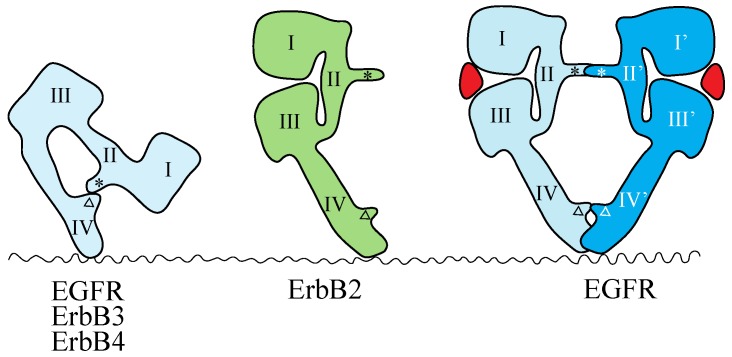
Schematic representations of the structures of the extracellular regions of the ErbB family. EGFR, ErbB3 and ErbB4 adopt the tethered conformation in the absence of ligand, while ErbB2 adopts an extended, or untethered, conformation that resembles the ligand-activated, dimerization-competent EGFR protomer in the ligand-bound form of the EGFR dimer, shown at the right. The ‘dimerization arm’ and ‘tethering arm’ are shown by an asterisk and an open triangle, respectively. Ligands are shown in red. Domains I–IV correspond to the domains shown in Figure 1. Not drawn to scale.

The crystal structures of the extracellular region of unliganded ErbB receptors [142,143,144,145] and of ligand-bound EGFR [146,147] have revealed large conformational changes that are crucial for ligand-induced dimerization of the receptor extracellular regions. An intramolecular tether is observed in the extracellular region of unliganded EGFR, ErbB3 and ErbB4. The ‘dimerization arm’ of II/CR1 is buried and interacts with loops at the C-terminal end of IV/CR2 to form an ‘auto-inhibited’ conformation (Figure 2) [142,143,144]. In the ligand-bound form, the I-II/L1-CR1 unit is rotated and moved away from the IV/CR2 domain, so as to be stabilized in an extended configuration in which the II/CR1 and IV/CR2 loops of the EGFR are both exposed and positioned to interact with a second partner to form the 2:2 back-to-back complex (Figure 2) [146,147]. Each ligand molecule is clamped between the I/L1 and III/L2 domains of the same EGFR molecule. The ligand-free ‘tethered’ configuration and the ligand-bound ‘extended’ configuration are mutually exclusive. In addition, parts of Domain IV/CR2 are thought to come close (or into direct contact) at the dimer interface based on both biochemical studies [148] and modeled structures [143]. In a dimer of EGFR extracellular regions, all intermolecular contacts are mediated by the receptor [146,147], making EGFR unique among RTKs with known ligand-bound structures. In all other such cases, the ligand contributes directly to the dimer interface [7]. 

Mutations that weaken or eliminate the II–IV/CR1–CR2 interaction indicate that untethering alone is insufficient for EGFR activation and that ligand binding is essential for the correct positioning of the two receptors to achieve full kinase activation [149]. The extracellular region of ErbB2, which does not have a known ligand, exists in the ‘extended’ configuration (Figure 2) [150,151], poised to interact with the ligand-bound form of the extracellular region of other EGFR family members. This suggests that ErbB2 can function as a heterodimer partner for the other ErbB family members, as described above. 

The recent structures of the EGFR kinase in an apparently inactive, symmetric dimer [152,153,154] and an active, asymmetric dimer [153] provide insight into the structural underpinnings of these conformational changes. In the inactive, symmetric conformation, the helix αC of the N-terminal lobe of the kinase domain is rotated outward with respect to its conformation in the active state, and the centrally located activation loop is tightly packed inside the active site in a way that blocks the binding of peptide substrates. Upon activation, the αC helix rotates toward the active site, resulting in an open conformation of the activation loop that is compatible with the binding of substrate peptides [152,153,155]. In the active, asymmetric kinase domain dimer, the C-terminal lobe of the ‘activator/donor’ kinase contacts the N-terminal lobe of the adjacent ‘receiver/acceptor’ kinase and promotes conformational changes that activate the ‘receiver/acceptor’ kinase. Thus, ligand binding is likely to dissociate the inactive, symmetric kinase dimer, resulting in the active, asymmetric kinase dimer, in which the ‘activator/donor’ kinase activates the adjacent ‘receiver/acceptor’ kinase. This type of interaction is reminiscent of the activating interaction between cyclins and the N-terminal lobes of cyclin-dependent kinases and was suggested to be conserved in other ErbBs, based on their amino acid sequence similarity [153,156]. In the active, asymmetric dimer, the N-terminal portions of the intracellular JM region (referred to as JM-A) of the receiver/acceptor and the activator/donor kinases are likely to interact, and the residues important for this interaction are conserved among the four ErbB family members. The C-terminal portion of the JM region (referred to as JM-B or JMAD) of the ‘receiver/acceptor’ kinase interacts with the C-terminal lobe of the ‘activator/donor’ kinase domain in a latching interaction [152,157]. 

**Figure 3:**
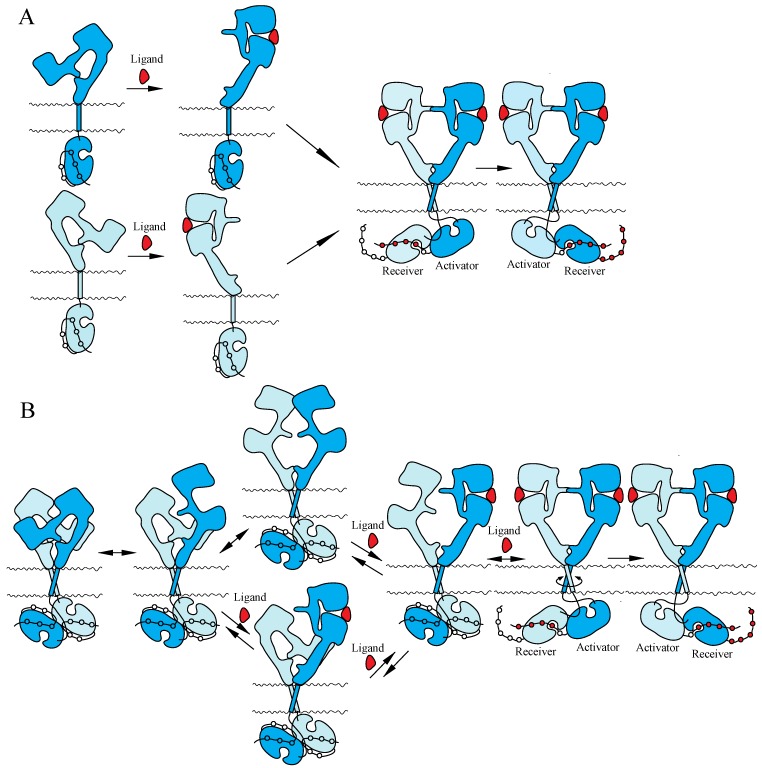
Models for ligand-induced activation of the ErbB family. (**A**) ‘Dimerization’ model. (**B**) ‘Rotation/twist’ model. For the explanation of the models, see the main text. Not drawn to scale.

Two mutually exclusive ‘dimerization’ and ‘rotation/twist’ models for ligand-induced activation of the ErbB receptors have been proposed, based on the monomeric and dimeric structures of inactive ErbBs prior to ligand binding, respectively (Figure 3). Both of the models are consistent with the crystallographic structures with and without bound ligand described above. In the ‘dimerization’ model [11,14,115], the receptor is proposed to exist as a monomer at the cell surface prior to ligand binding. Ligand binding to the extracellular I–III/L1–L2 domains of the tethered form releases the interaction between II/CR1 and IV/CR2 and exposes the ‘dimerization arm’ of the II/CR1 domain in its extended configuration. This exposed ‘dimerization arm’ interacts with the exposed ‘dimerization arm’ of the other receptor to form a dimer, in which two intracellular kinase domains assume the asymmetric active structure. In contrast, the ‘rotation/twist’ model [15,22] predicts that the receptor exists as a dimer at the cell surface, in which the extracellular regions have the tethered structure and the intracellular kinase domains have the symmetric, inactive dimeric structure. Upon ligand binding to untethered extracellular regions of the receptor dimer, two exposed ‘dimerization arms’ interact with each other to form extended configurations. This conformational change, from untethered to extended, induces the rotation/twist of TM segments [15] that rearrange the inactive, symmetric kinase domain dimers to take the active, asymmetric conformation. In the asymmetric kinase dimers, the ‘activator/donor’ kinase activates the adjacent ‘receiver/acceptor’ kinase, as described above. 

### 4.2. NT Receptor Family

Early work on PC12 cells provided the initial evidence that NGF mediated its effects by binding to the TrkA receptor, inducing receptor dimerization and autophosphorylation [58,70,158]. Because NT ligands exist in solution as stable, non-covalently associated homodimers [159,160,161], it is thought that a single NGF dimer acts as a bridge to induce the dimerization of two TrkA monomers [162]. This model was supported by the crystal structure of NGF bound to the ligand-binding immunoglobulin domains of TrkA, which has been shown to have a symmetric 2:2 stoichiometry in the complex [86]. This ‘dimerization’ model suggests that the high local membrane density of Trk receptor proteins may promote spontaneous activation in the absence of NTs under scenarios of overexpression. Recent studies using chemical crosslinking and reversible firefly luciferase fragment complementation assay, however, demonstrate that all the TrkA and TrkB receptors exist as pre-formed, yet inactive, dimers at the surface of living cells [26,27]. These results are consistent with the recent crystal structures of inactive, symmetric kinase domain dimers of TrkA and TrkC [81,163]. Therefore, the ligand-induced dimerization model needs to be re-evaluated.

NT-dependent p75^NTR^ activation involves the association of an NT dimer with repeats of a 40-amino acid, cysteine-rich domain of the two extracellular regions of a p75^NTR^ dimer [130]. As described above, an unusual feature of the p75^NTR^ structure is the existence of a disulfide-linked p75^NTR^ dimer, formed through cysteine residues within its TM segment. This disulfide linkage is required for effective NT-dependent signaling by p75^NTR^ [128]. Recent studies propose a model in which NT binding causes the two extracellular regions of p75^NTR^ dimers to move closer together, forcing the intracellular regions to splay apart in a snail-tong-like, or scissors-like, motion centered on the disulfide bond and permitting the association of the intracellular regions with the signaling adapter proteins, NRIF and TRAF6 [128,164]. 

### 4.3. IR Family

IR receptors are present as disulfide-linked (αβ)_2_ homodimers at the cell surface, as described above. Ligand binding to the extracellular region of the receptors induces conformational changes in their structures and activates their intrinsic tyrosine kinase activity. Two scenarios can be envisioned for the inactive state prior to ligand binding. First, the intracellular kinase domains are spatially separated and, thus, cannot undergo *trans*-phosphorylation. Second, the kinase domains are arranged to prevent *trans*-phosphorylation (perhaps as an inactive dimer). A partially activating point mutation (Y984A) in the intracellular JM region of IR [165] is more consistent with the latter mechanism [166]. In the crystal structure of the IR kinase domain [167], the unphosphorylated activation loop adopts an autoinhibitory configuration in which the second of the three activation-loop tyrosines is bound in the kinase active site through hydrogen bonding to residues in the catalytic loop. This inhibitory conformation is observed in IGF-IR, as well [168]. Upon IR *trans*-phosphorylation, the phosphorylated activation loop is stabilized in a conformation that is optimized to bind substrates for catalysis [166]. Despite the recent structural elucidation of the insulin-bound IR extracellular region [169], the mechanism of insulin-initiated transmembrane signaling remains largely elusive. 

## 5. Cooperative Ligand Binding

Scatchard analysis of the binding of EGFR and IR with EGF and insulin, respectively, yields concave-up or curvilinear plots that indicate the presence of two classes of binding sites. It has long been thought that EGFR exists as both monomers and dimers at the cell surface, with low and high affinity, respectively [11,170]. However, the heterogeneity in EGF-binding affinities has recently been proposed to arise from negative cooperativity in the interaction between EGF and its receptor [171,172]. Analysis of insulin binding to IR also reveals curvilinear (concave-up) Scatchard plots, which has also been explained by a negative cooperative interaction between insulin and IR [173]. 

### 5.1. ErbB Family

EGF binding to cell-surface receptors was first reported over 30 years ago [174,175]. EGF binding has been characterized by either negative cooperativity or heterogeneity of sites and has traditionally been interpreted to suggest the existence of two classes of EGF-binding site at the cell surface [170,175]. In this analysis, a high-affinity class accounts for ~10% of receptors with a dissociation constant (*K*_d_) of ~10^−10^ M and a low-affinity class with a *K*_d_ of ~10^−9^ M. It is generally believed that the mitogenic actions of EGF are mediated through the high affinity site [176,177,178,179]. The nature of the high- and low-affinity sites is still under debate. EGFR phosphorylation at Thr-654 by protein kinase C reduces the number of high-affinity EGF-binding sites at the cell surface [180], and deletions from the intracellular region of EGFR are likely to prevent the receptor from forming high-affinity sites [181,182]. However, models demonstrating that the high- and low-affinity sites represent EGFR dimers and monomers, respectively, cannot explain the observed binding characteristics. All such models lead to positive cooperativity with concave-down, not concave-up, Scatchard plots [183]. Biophysical analysis of the isolated extracellular region of human EGFR suggested a positive cooperative interaction between EGF and EGFR [184]. The concave-up Scatchard plots observed at the cell surface could only be explained by invoking an ‘external site’ that independently stabilizes a fraction of receptor molecules in a high-affinity dimeric state [181,185,186]. Heterogeneities in receptor density at the cell surface have been employed by others to explain the observed concave-up Scatchard plot [187]. A requirement for negative cooperativity is that EGFR dimers bind one EGF. Binding of a second EGF must occur with lower affinity than the first. Indeed, a recent study of ^125^I-labelled EGF binding to EGFR at the cell surface [171,172] yielded a model characterized by negative cooperativity that had previously been predicted [183]. The crystal structures of the *Drosophila* EGFR extracellular region suggest negative cooperativity between EGFR and its ligand [188]. Consistent with this, a recent study suggests that a single ligand may be sufficient to activate EGFR dimers [189]. In contrast, there is evidence showing that binding of two EGF molecules is required for EGFR autophosphorylation [190]. An electron microscopy study of nearly a full-length EGFR bound with EGF also showed no evidence of negative cooperativity [191]. 

There is evidence that interaction between the ‘tethering’ arms (Residues 561–585 of Domain IV/CR2 in the extracellular region; Figure 2) in the EGFR dimer is essential for negative cooperativity in EGF binding [192]. Breaking the disulfide bond between Cys-558 and Cys-567 of the tethering arm of the Domain IV/CR2 in the extracellular region of EGFR through double alanine replacements or deleting the loop entirely decreased negative cooperativity in EGF binding. Deletion of the loop between Cys-571 and Cys-593 of the tethering arm also abrogates negative cooperativity. These results demonstrate that the tethering arm plays an important role in supporting cooperativity in ligand binding and suggest that the tethering arm contributes to intersubunit interactions within the EGFR dimer prior to ligand binding [192]. 

The intracellular JM region is divided into two segments, termed JM-A, which includes Residues 645–664, and JM-B/JMAD, which includes Resides 665–682, as described above [152,157]. In the crystal structure of the asymmetric dimer of the intracellular region [157], the JM-A segment appears as a helix oriented away from the kinase domains. Furthermore, NMR and mutational analyses of the JM-A segment suggest that this region may form an anti-parallel helical dimer that functions as a clasp to stabilize the asymmetric dimer [152]. Binding of EGF to its receptor is negatively cooperative [171], and the intracellular JM region is required for this allosteric regulation of ligand binding [172]. Internal deletion of the JM-A segment results in the complete loss of all cooperativity in EGF binding [193]. This suggests that JM-A plays a role in the ligand binding properties of the receptor. In the anti-parallel helical dimer of JM-A in the receptor dimer, Glu-663 and Glu-666 are predicted to be involved in interhelical salt bridges that would stabilize the helical dimer. When these ionic interactions are removed by mutation, negative cooperativity in EGF binding to EGFR is abrogated [193]. This again suggests that the proposed helical dimer could contribute to negative cooperativity in EGFR. It has long been recognized that phosphorylation of EGFR at Thr-654 leads to a decrease in the affinity of EGF and a loss of EGF-stimulated receptor autophosphorylation [174,175,194]. Phorbol 12-myristate 13-aceatte (PMA)-induced phosphorylation of Thr-654 leads to the complete loss of cooperativity [193]. These results suggest that the JM-A segment represents an important structural component underlying negative cooperativity in EGFR.

### 5.2. NT Receptor Family

Steady-state binding experiments have demonstrated that TrkA has a single binding site for NGF with a *K*_d_ of 10^−9^ M, a value reflecting low affinity binding, similar to the p75^NTR^ interaction [58,71]. This is consistent with the crystallographic structure of two Ig2 domains bound with an NGF dimer [86]. Reconstitution experiments by transient transfection into heterologous cells indicate that high-affinity NGF binding requires co-expression of both p75^NTR^ and TrkA [71,195,196,197], while p75^NTR^ has a negative effect on TrkB tyrosine autophosphorylation in response to BDNF and NT-4/5 and no effect on TrkB and TrkC activation in response to NT-3 [198]. Over the past two decades, a number of mechanistic models of the functional interactions between p75^NTR^ and TrkA have been proposed [199], which include the formation of a classic 1:1 heterodimer with higher affinity than the individual receptors [71,195], and the ligand-passing model in which p75^NTR^ first binds to NGF before releasing the ligand for TrkA to bind [195,199]. However, these models are inconsistent with the observation that the extracellular ligand-binding domain of p75^NTR^ is not required to create high-affinity NGF binding sites [200]. A recent observation also indicates that the p75^NTR^ intracellular domain fragment, but not the full-length p75^NTR^, enhances NGF binding to TrkA [133]. These observations suggest that small intracellular peptides of p75^NTR^ interact with intracellular regions of TrkA (perhaps in its dimeric form) to induce conformational changes of the receptor’s intracellular region. This may, in turn, induce conformational changes of the extracellular regions that lead to a high affinity conformation of the receptor (dimer) for NGF binding. 

### 5.3. IR Family

IR family receptors are pre-formed disulfide-linked (αβ)_2_ dimers, and *trans*-phosphorylation occurs within the dimer rather than via higher-order oligomerization [201]. Analysis of insulin binding to IR reveals concave-up (curvilinear) Scatchard plots and negative cooperativity, indicating that only one insulin molecule binds to one (αβ)_2_ dimer [173,202]. Each monomer in the IR dimer is thought to contain two separate ligand-binding regions, Site 1 and Site 2, on one monomer, and Site 1’ and Site 2’, on the other. Binding of ligand to Site 1 of one monomer is then followed by ‘cross-linking’ of the bound ligand to the second site (Site 2’) of the alternate monomer [203]. Within this model, negative cooperativity results from subsequent ligand binding to the alternate Site 1’/Site 2 pair and concomitant release of ligand at the initial Site 1/Site 2’ pocket. This model suggests that a pair of ligands cannot cross-link Site 1/Site 2’ and cross-link Site 1’/Site 2 simultaneously [204]. This model has recently been refined by assuming that the formation of the high-affinity cross-link is enabled by harmonic oscillation of the receptor [205,206].

In support of this hypothesis, purified αβ monomers prepared by the mild reduction of interhalf disulfide (Class I disulfide) bonds show only low affinity binding with a stoichiometry of one insulin per αβ monomer, whereas purified (αβ)_2_ dimers exhibit negative cooperativity and only one high affinity insulin-binding site [207,208]. Double probe analysis using two different insulin analogues showed that only one analogue could bind to the receptor with high affinity at a time [209,210]. Unlike the full-length receptors, soluble extracellular regions show only low-affinity ligand binding, unless they are C-terminally tethered by transmembrane anchors, Fc domains or leucine zippers [211]. In addition to negative cooperativity, positive cooperativity for IR binding has also been reported at low insulin concentrations [212]. That is, receptor occupancy seems to enhance the binding of insulin to its receptor at low ligand levels. 

## 6. Concluding Remarks

It is now clear that ErbB receptors are allosteric enzymes, indicating that they are likely to dimerize in the absence of ligand. Indeed, a wide variety of studies demonstrate the ErbB receptors exist as dimers at the surface of living cells prior to ligand binding [15,17,18,19,20,21,22,23,24,25,28,29]. NT receptors also exist as covalently-linked p75^NTR^ or non-covalently-linked pre-formed TrkA and TrkB dimers prior to ligand binding [26,27,128]. These dimeric structures are reminiscent of IR, which is a covalently-linked pre-formed dimer that nonetheless requires insulin binding for activity [203]. Cooperativity long observed in the interaction between insulin and IR is now also observed in the EGF-EGFR interaction [171]. These observations suggest that EGFR and IR may be activated by similar mechanisms. Indeed, an early study on signaling by IR::EGFR chimeras argued that the regulatory mechanisms of these two receptor classes are closely related [213]. Thus, RTKs may be activated by cognate ligand binding through similar mechanisms.
